# Treatment of vaginal candidiasis for the prevention of preterm birth: a systematic review and meta-analysis

**DOI:** 10.1186/s13643-015-0018-2

**Published:** 2015-03-21

**Authors:** Christine L Roberts, Charles S Algert, Kristen L Rickard, Jonathan M Morris

**Affiliations:** Kolling Institute of Medical Research, University of Sydney, St Leonards, NSW Australia

**Keywords:** Pregnancy, Preterm birth, Premature infant, Candida, Candidiasis, Yeasts, Randomised controlled trial, Met-analysis

## Abstract

**Background:**

Recognition that ascending infection leads to preterm birth has led to a number of studies that have evaluated the treatment of vaginal infections in pregnancy to reduce preterm birth rates. However, the role of candidiasis is relatively unexplored. Our aim was to undertake a systematic review and meta-analysis to assess whether treatment of pregnant women with vulvovaginal candidiasis reduces preterm birth rates and other adverse birth outcomes.

**Methods:**

We undertook a systematic review and meta-analysis of published randomised controlled trials (RCTs) in which pregnant women were treated for vulvovaginal candidiasis (compared to placebo or no treatment) and where preterm birth was reported as an outcome. Trials were identified by searching the Cochrane Central Register of Controlled Trials, Medline and Embase databases to January 2014. Trial eligibility and outcomes were pre-specified. Two reviewers independently assessed the studies against the agreed criteria and extracted relevant data using a standard data extraction form. Meta-analysis was used to calculate pooled rate ratios (RR) and 95% confidence intervals (CI) using a fixed-effects model.

**Results:**

There were two eligible RCTs both among women with *asymptomatic* candidiasis, with a total of 685 women randomised. Both trials compared treatment with usual care (no screening for, or treatment of, asymptomatic candidiasis). Data from one trial involved a post-hoc subgroup analysis (*n* = 586) of a larger trial of treatment of 4,429 women with asymptomatic infections in pregnancy and the other was a pilot study (*n* = 99). There was a significant reduction in spontaneous preterm births in treated compared with untreated women (meta-analysis RR = 0.36, 95% CI = 0.17 to 0.75). Other outcomes were reported by one or neither trial.

**Conclusions:**

This systematic review found two trials comparing the treatment of asymptomatic vaginal candidiasis in pregnancy for the outcome of preterm birth. Although the effect estimate suggests that treatment of asymptomatic candidiasis may reduce the risk of preterm birth, the result needs to be interpreted with caution as the primary driver for the pooled estimate comes from a post-hoc (unplanned) subgroup analysis. A prospective trial with sufficient power to answer the clinical question ‘does treatment of asymptomatic candidiasis in early pregnancy prevent preterm birth’ is warranted.

**Systematic review registration:**

PROSPERO CRD42014009241

## Background

Preterm birth is a major pregnancy complication affecting 5% to 18% of births worldwide [1,2]. Infants born preterm are at increased risk of death, significant neonatal complications, long-term adverse health outcomes and developmental impairment [3-5].

Preterm birth (birth before 37 completed weeks of gestation) results from either spontaneous onset of labour (including preterm prelabour rupture of the membranes) or a clinical decision that planned birth should occur because of pregnancy complications. The cause of spontaneous preterm birth is often unknown, but intrauterine infection is implicated in up to 40% [4,6,7]. The likely pathway to intrauterine infection is ascending genital tract infection [6-9]. Genital tract infection is more frequent among women with spontaneous preterm births at lower gestational ages [7,10]. Importantly, infection may occur before or early in pregnancy, may be asymptomatic and may remain undetected [7,11].

The role of infection in preterm birth is thought to be a chronic process, with early pregnancy a period of vulnerability to establishment of inflammatory responses that may be the trigger for preterm parturition [6,9,11]. Organisms detected in the uterus before membrane rupture are typically of low virulence, probably accounting for both the chronicity of intrauterine infections and the frequent absence of overt clinical signs of infection [6,8].

Pregnancy increases the frequency of vaginal *Candida* colonization [12]. This is thought to be the consequence of increased levels of circulating oestrogens and deposition of glycogen and other substrates in the vagina during pregnancy [12]. *Candida* colonisation may disrupt normal vaginal flora so that there is a decrease in lactobacilli and an increase in proinflammatory organisms [9,13]. However, few studies have assessed the associations between candidiasis and preterm birth. A systematic review of treatment trials for symptomatic candidiasis during pregnancy assessed ‘cure’ (negative culture or symptom relief posttreatment) but not pregnancy outcomes [14].

Studies utilising population-based data from Hungary reported that vaginal clotrimazole treatment of candidiasis during pregnancy was associated with a 34% to 64% reduction in the prevalence of preterm birth [15-17]. In contrast, two cohort studies found no significant association between preterm birth and moderate to heavy growth of *Candida* species among women at 22 to 30 weeks gestation [18,19]. Therefore, our aim was to undertake a systematic review and meta-analysis to assess whether the treatment of pregnant women with vulvovaginal candidiasis reduces preterm birth rates and other adverse birth outcomes. The importance of reducing preterm birth rates warranted performing a meta-analysis of randomised clinical trials, which might amass sufficient statistical power to provide clear evidence about a possible protective effect.

## Methods

### Search strategy

The study procedure and outcomes were pre-specified [20]. We identified relevant studies by searching the Cochrane Central Register of Controlled Trials, Medline and Embase from database inception through 31 January 2014. There were no language restrictions. The database searches were supplemented by hand-searching the reference lists of relevant reviews and potentially eligible studies. Search terms (using keywords and Medical Subject Headings (MeSH), all exploded) included (‘candida’ or ‘candidiasis’ or ‘candidosis’ or ‘yeasts’) and (‘pregnancy’ or ‘preterm/premature birth’) and ‘antifungal agents’. Conference and meeting abstracts were not included, and no attempt was made to identify unpublished studies or contact the authors of published studies.

### Eligibility criteria

Randomised controlled trials (RCT) in which pregnant women were treated for symptomatic or asymptomatic vulvovaginal candidiasis and where preterm birth was reported as an outcome were the pre-specified eligibility criteria [20]. Only RCTs that compared treatment (imidazoles or other proven therapeutic agents) with placebo or no intervention were of interest. Quasi-randomised designs, such as alternate allocation or the use of medical record numbers, were not eligible. Mycologically confirmed diagnoses of vulvovaginal candidiasis (i.e. a positive culture and/or microscopy for yeast) were required.

### Study selection

The titles and abstracts of all potential studies identified for inclusion as a result of the search strategy were independently assessed for inclusion by two reviewers. The two reviewers also assessed the full papers of potentially eligible studies or where eligibility was unclear. Discrepancies at both stages of study selection were resolved through discussion.

### Risk of bias

The two review authors also independently assessed the risk of bias (as low, high, or unclear) for each study using the following pre-specified criteria: random sequence generation, allocation concealment, blinding of participants and personnel, blinding of outcome assessment and completeness of outcome data [20]. Again, discrepancies were resolved through discussion.

### Primary outcome

Preterm birth (<37 completed weeks of gestation) following spontaneous onset of labour or following preterm prelabour rupture of membranes.

### Secondary infant outcomes

1. Any birth before 37 weeks
2. Medically indicated birth (by labour induction or prelabour caesarean section) before <37 weeks
3. Birth before 32 weeks
4. Birth weight less than the tenth percentile for gestational age
5. Birth weight <2,500 g
6. Apgar score of less than 7 at 5 min
7. Respiratory distress syndrome
8. Use of mechanical ventilation
9. Duration of mechanical ventilation
10. Intraventricular haemorrhage
11. Retinopathy of prematurity
12. Chronic lung disease
13. Necrotising enterocolitis
14. Perinatal mortality (stillbirth or neonatal death)
15. Admission to neonatal intensive care unit
16. Neonatal length of hospital stay
17. Breastfeeding

### Secondary maternal outcomes

1. Preterm prelabour rupture of the membranes
2. Spontaneous pregnancy loss <20 weeks gestation,
3. Mode of birth
4. Duration of maternal hospitalisation at the time of birth
5. Maternal views/satisfaction with the therapy
6. Maternal anxiety

### Data extraction

Data were extracted independently by the two reviewers, and disagreements were resolved by discussion. A standard data extraction form was used to extract data on study characteristics, methods and study results. All data on study results were entered into an Excel Spreadsheet.

### Data synthesis

Characteristics, main findings and risk of bias assessment were tabulated for each study. The raw data presented in the included studies were used to determine the outcome rates for intervention and control groups of each study. Where data were missing (incomplete follow-up on all women), the results reported in the studies as the numerator (no information on those lost to follow-up) and denominator (all women randomised) were used; no imputation of outcomes was made. There were two pre-specified subgroup analyses for the primary outcome: symptomatic and asymptomatic candidiasis, and commencing treatment before 20 weeks gestation versus after 20 weeks gestation. For each dichotomous outcome of interest within individual studies, relative risks (RR) and 95% confidence intervals (CIs) were calculated according to the intention to treat. The assumption of homogeneity of treatment effect between studies was assessed using Cochran’s Q test statistic and the I^2^ test. Meta-analysis was used to calculate pooled risk ratios (RR) and 95% confidence intervals (CI) using a fixed-effects model (Mantel-Haenszel), unless the assumption of homogeneity was rejected (*P* < 0.1) when a random effects model would be used. Statistical analyses were performed using the ‘metan’ command in STATA (STATA statistical software version 11.0, STATA, College Station, USA).

## Results

### Literature search results

**Figure 1:**
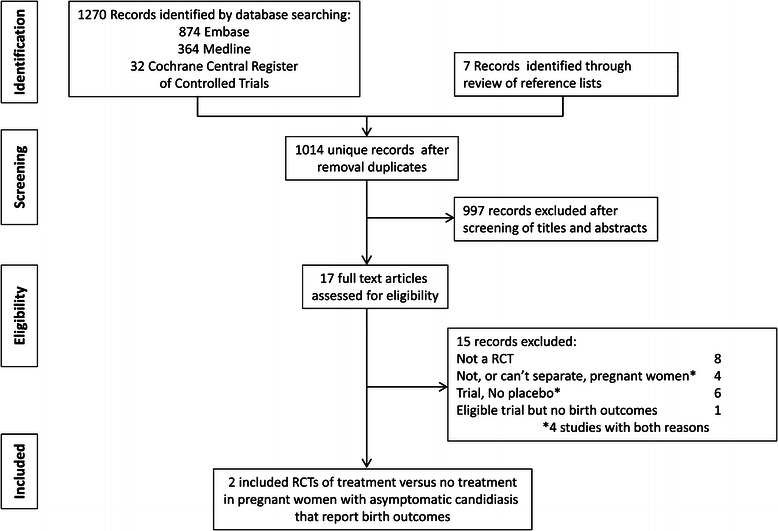
**Summary of evidence search and selection.**

A total of 1,014 unique articles were identified (Figure 1). Of these 17 underwent full review as potentially eligible or where the eligibility was unclear from the title and abstract [21-37]. Three papers compared treatment versus placebo or no intervention for pregnant women with candidiasis [29,35,37] but only two had the outcome of preterm birth and were eligible for inclusion (Table 1) [29,35].

**Table 1:** **Characteristics of randomised controlled trials assessing treatment of vaginal candidiasis to prevent preterm birth**

Study	Study period and location	Study population	Study size (Candidiasis)	Intervention	Comparison	Available outcomes among women with candidiasis	Funding and competing interests
Kiss et al.	2001 to 2002, 25 non-hospital-based obstetricians Vienna, Austria	Women with singleton pregnancies, 15^0^ to 19^6^ weeks gestation, no symptoms of vaginal infection, bleeding or contractions, Mean age [SD]: 28.9 [±5.6], 48% primipara 98% white ethnicity, Carriage rate of asymptomatic candidiasis: 14.1%	586, 294 randomised to treatment 292 randomised to usual care	Vaginal clotrimazole 0.1 g for 6 days	Usual care (vaginal culture result not revealed, no treatment)	Spontaneous preterm birth (<37 weeks gestation)	‘Healthy Austria’ (‘Fonds GesundesÖsterreich’) grant PNr.205/V/12 and Federal Ministry of Education, Science, and Culture grant, GZ 70.069/1-Pr4/2000, no competing interests declared.
Roberts et al.	2008 to 2009, single tertiary obstetric hospital, Sydney, Australia	Women with singleton pregnancies, 12^0^ to 19^6^ weeks gestation, no symptoms vaginal infection, mean age [SD]: 32.2 [±54.4], 45% primipara, ethnicity not reported, carriage rate of asymptomatic candidiasis: 19.6%	99, 50 randomised to treatment, 49 randomised to usual care	Vaginal clotrimazole 0.1 g for 6 days	Usual care (vaginal culture result not revealed, no treatment)	Spontaneous preterm birth (<37 weeks gestation); any preterm birth; pregnancy complications (gestational diabetes; antepartum haemorrhage); mode of delivery (spontaneous vaginal, instrumental caesarean section), birth weight (<2,500, 2,500 to 3,999, ≥4,000 g); nursery admission.	One author supported by an Australian National Health and Medical Research Council Fellowship. No competing interests declared.*

SD, standard deviation; *three authors of this paper are also authors of this systematic review.

### Characteristics of included studies

Both studies included women with *asymptomatic* vaginal candidiasis and both compared treatment with clotrimazole to no treatment (Table 1) [29,35]. Spontaneous preterm birth was the primary outcome for both studies. As spontaneous preterm birth was the only outcome available from both studies, none of the other planned meta-analysis outcomes could be assessed. There were no relevant studies assessing *symptomatic* candidiasis where preterm birth was the outcome.

The aim of the study by Kiss and colleagues was to assess whether general screening for, and treatment of, asymptomatic vaginal infections (bacterial vaginosis, candidiasis and/or trichomoniasis) was effective in reducing the rate of preterm birth and late miscarriage [29]. Women who were culture positive for any of the three conditions (*n* = 4,429) were randomised to treatment (appropriate to the organism: clindamycin, clotrimazole and/or metronidazole, respectively) or to usual care (culture result not revealed and no treatment). There was no pre-specified subgroup analysis by infection type so the information on treatment of asymptomatic candidiasis from this trial was extracted from the published paper post-hoc.

Drawing on post-hoc subgroup analyses of the Kiss trial, Roberts and colleagues undertook a pilot study with the specific aim of assessing treatment of asymptomatic candidiasis to prevent preterm birth [35]. The study design was essentially the same although the eligibility criteria were limited to women with asymptomatic candidiasis.

The asymptomatic *Candida* colonisation rate was 14.1% (15 to 19 weeks gestation) in the Kiss et al. study and 19.6% (12 to 19 weeks gestation) in the Roberts et al. study. Kiss et al. reported that women were to be retested, and if necessary retreated, at 24 to 27 weeks. However, overall, only 22% of women in the entire treatment arm had a follow-up gram stain and of these 27% still had a vaginal infection present, including 78 (27%) with candidiasis, all of whom were retreated. Roberts et al. report a posttreatment colonisation rate of 48% on average 10 weeks after recruitment but asymptomatic women were not offered further treatment.

### Risk of bias

Both studies utilised computer random number generation and central randomisation procedures. In the Kiss et al. study, women who were randomised to clotrimazole treatment (and their obstetricians) were not blinded to their treatment allocation. However, the untreated group (93% of women screened) included both women without infections and those with asymptomatic infections who were randomised to usual care. Clinicians and women were blinded to the colonisation-status within this group. Roberts et al. used a similar method but women allocated to clotrimazole treatment were notified and treated by the study personnel. So, although the women in the treatment arm were not blinded, clinicians were blinded to treatment allocation unless it was revealed during the subsequent pregnancy management. Like the Kiss et al. study, the untreated group (90% of participants) included women with and without asymptomatic candidiasis and the clinicians and women were blinded to this information. This partial blinding of participants and personnel to exposure status was considered unlikely to affect results. Furthermore, the assessment of outcomes from medical records was blinded and gestational age determination is not subjective (based on ultrasound dating and date of birth). The candidiasis subgroup analysis in the Kiss et al. study was not pre-specified but extracted post-hoc from the published paper. Furthermore, Kiss et al. did not report loss to follow-up and exclusions by treatment arm or infection subgroups so the number of women with candidiasis for whom outcome data were missing could not be determined. However, overall 3.2% women were lost to follow-up and there were 3.0% post-randomisation exclusions (1.5% multiple pregnancies; 1.5% did not fulfil the inclusion criteria). The follow-up rate was 99% in the Roberts et al. study (outcome information was missing for one woman) with no post-randomisation exclusions.

For both studies, the risk of bias was considered low for all aspects assessed: random sequence generation, allocation concealment, blinding of participants and personnel, blinding of outcome assessment and completeness of outcome data. However, neither study had a published protocol so it is possible that there was selective choice for reporting of secondary outcomes. Acknowledged funding for the trials was from government sources, and there is no suggestion (although not explicitly stated) that the trial treatment was provided by a pharmaceutical company (Table 1).

### Data synthesis

**Figure 2:**
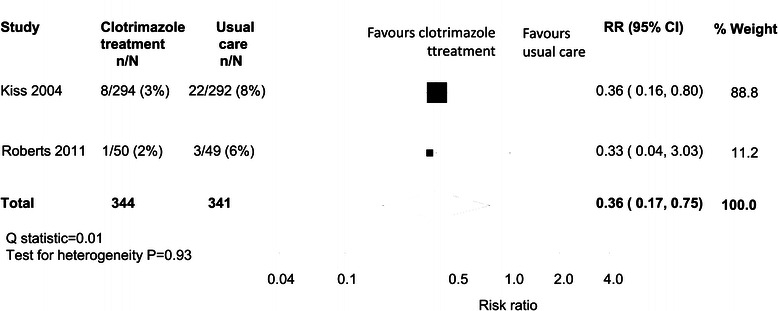
**Meta-analysis of spontaneous preterm birth among women with asymptomatic candidiasis: clotrimazole versus usual care.**

The only outcome available from both studies was spontaneous preterm birth. Meta-analysis showed an overall reduction in spontaneous preterm birth (RR = 0.36, 95% CI = 0.17 to 0.75) with similar point estimates from both studies but little contribution (and very wide confidence intervals) around the estimate from the pilot study by Roberts et al. (Figure 2). While Roberts et al. reported on subsequent pregnancy complications, labour induction, mode of delivery, birth weight and nursery admissions, no other outcomes were available from the Kiss trial.

## Discussion

This systematic review found two trials comparing the treatment of asymptomatic vaginal candidiasis in pregnancy with usual care (no screening and no treatment of asymptomatic vaginal candidiasis) for the outcome of preterm birth. The findings provide support for the hypothesis that treatment of asymptomatic candidiasis may reduce the risk of preterm birth. Although the two studies had similar methods, treatment regimens and findings among general maternity populations in different countries, the result needs to be interpreted with caution as the primary driver for the pooled estimate is a post-hoc subgroup analysis of the Kiss trial. We believe that the meta-analysis result supports the need for a larger trial that specifically addresses the question of whether the treatment of asymptomatic candidiasis early in pregnancy can reduce the risk of spontaneous preterm birth. If a simple, inexpensive intervention is demonstrated to reduce spontaneous preterm birth, this would change current maternity care internationally. A significant reduction in preterm birth would not only reduce perinatal mortality and morbidity, but also have major resource implications, such as reduced need for neonatal intensive care and childhood hospitalisations.

The two trials reported different colonisation rates of asymptomatic candidiasis (14.1% and 19.6%) [29,35]. This reflects population baseline characteristics and slightly varying gestational age ranges for recruitment. Other studies report colonisation rates that range from 14% to 38% for symptomatic candidiasis at 22 to 30 weeks gestation but do not report asymptomatic rates [18,19,38]. Some of the population risk factors for candidiasis are also risk factors for preterm birth including African-American women, low socioeconomic status, smoking, maternal medical conditions and bacterial vaginosis [16,18,29].

Both trials included in the meta-analysis used a similar design, described by Roberts et al. as a Prospective, Randomised, Open-label, Blinded-Endpoint (PROBE) design. PROBE designs have been used in cardiovascular disease trials [39-46], and the two trials in this review may be the first obstetric trials to use this design. Features include strict randomisation and allocation concealment procedures, and blinding of those assessing the trial endpoints [41]. The drug interventions are typically commercially available as indicated in the Roberts trial [35]. Consequently, as the treatment protocol adheres closely to routine clinical practice, the results from a PROBE design may be more generalisable to the pragmatic management of patients than double-blind, placebo-controlled trials [41,45]. A potential disadvantage of a placebo-controlled trial for answering this preterm birth prevention question is that a vaginally administered placebo may be biologically active as it would have to contain an alcohol preservative that could have an independent effect on vaginal flora [35].

Clotrimazole, the treatment used in both the included studies, is classified as a category A drug meaning it has been used by a large number of pregnant women and women of childbearing age without any proven increase in the frequency of malformations or other direct or indirect harmful effects on the fetus having been observed [47]. Large population-based studies have not demonstrated risks to the fetus following exposure to clotrimazole in pregnancy [48]. Randomised trials of treatment of symptomatic candidiasis in pregnancy provide evidence for the use of topical imidazoles (such as clotrimazole), rather than nystatin or hydrargaphen, for successful eradication of *Candida* from the vagina. Furthermore, the susceptibility of both *Candida albicans* and non-albicans *Candida* vaginal isolates to azole antifungal agents, such as clotrimazole, supports the continued practice of azole antifungal agents for empirical therapy of *Candida* vaginitis [49].

Despite the interest in infection as a risk factor for preterm birth, we found only two trials (including a small pilot study) to contribute to this review. Perhaps as *Candida* is considered a vaginal commensal organism [13], the role of candidiasis in preterm birth has not been pursued with the same attention as bacterial vaginosis and other vaginal organisms [11,50-53]. However, it is also possible that some relevant studies that could change the finding of the meta-analysis in the direction of no association were missed as only controlled vocabulary search terms were used and the search was limited to published studies.

Rather than pregnancy outcomes, previous research has mostly focussed on the question of best treatment for eradicating *Candida* colonisation in pregnant women with symptomatic candidiasis. The availability of only two trials precludes the opportunity to explore issues like heterogeneity and any impact of reporting biases in sensitivity and subgroup analyses. Only one outcome (spontaneous preterm birth) was available from both studies, and future trials should consider other potential pregnancy outcomes and treatment side effects [54]. Although we identified 11 treatment trials of symptomatic candidiasis in pregnancy, all were published before 1985, only one compared treatment to placebo and none reported pregnancy outcomes, only the rate of *Candida* eradication [25,36,37,55-62]. Furthermore, the seven studies that reported gestational age at recruitment all included women who were too advanced in pregnancy to have an impact on preterm birth [25,36,37,56,58,60,62].

Inclusion in the meta-analysis of some women with missing outcome information is unlikely to change the conclusions. From the Kiss et al. trial, the extent of missing data on the outcome of preterm birth for women with candidiasis could not be determined but was approximately 3% among all women with asymptomatic infections. One woman (of 99) in the Roberts et al. trial was missing information on birth outcome. Only Roberts et al. reported other birth outcomes for the treated and untreated groups of women but this was limited by small event numbers: any preterm birth (RR = 0.65; 95% CI = 0.11 to 3.74), spontaneous vaginal birth (RR = 1.03; 95% CI = 0.64 to 1.64), instrumental vaginal birth (RR = 0.88; 95% CI = 0.39 to 1.98), caesarean section (RR = 1.09; 95% CI = 0.66 to 1.80), birth weight <2,500 g (RR = 0.98; 95% CI = 0.14 to 6.68) and nursery admission (RR = 1.31; 95% CI = 0.31 to 5.54). The remaining pre-specified outcomes were not reported in either of the included studies.

The rationale of the two included trials is that early treatment of vaginal infections is necessary for effective prevention of infection-related preterm birth, as early pregnancy is the period of greatest risk for the establishment of inflammatory responses to low virulence organisms that increase the risk of preterm birth [6-9]. Treatment later in pregnancy may have limited effect in preventing preterm parturition if the inflammatory responses are not fully reversible [4]. Importantly, treatment does not necessarily eradicate *Candida* in all women nor prevent recolonisation. Posttreatment ‘*Candida* eradication rates’ (assessed at 3 to 6 weeks) for *symptomatic* candidiasis in pregnancy range from 69% to 100% (five trials, median 88%) [14] and for *asymptomatic* candidiasis was 73% (assessed at 4 to 5 weeks) in the Kiss trial [29] and 52% (assessed at 10 weeks) in the Roberts trial [35]. However, it is not clear whether posttreatment colonisation represents persistent colonisation or recolonisation.

## Conclusion

The findings of this review, that treating asymptomatic candidiasis in early pregnancy may reduce spontaneous preterm birth rates, need to be interpreted with caution. The findings are based on only two published trials (a pilot study and a post-hoc subgroup analysis with data from 685 women who had 34 spontaneous preterm births) both using clotrimazole and both in asymptomatic women, and the post-hoc subgroup analysis was the primary driver for the pooled estimate. Furthermore, there were insufficient data on other important infant and maternal outcomes. This systematic review suggests that a trial with sufficient power to answer the clinical question ‘does treatment of asymptomatic candidiasis in early pregnancy prevent preterm birth’ is warranted.
